# Role of microRNAs in Predicting the Prognosis of Cervical Cancer Cases: A Systematic Review and Meta-Analysis

**DOI:** 10.31557/APJCP.2021.22.4.999

**Published:** 2021-04

**Authors:** Sasidharanpillai Sabeena, Nagaraja Ravishankar

**Affiliations:** 1 *Manipal Institute of Virology, Manipal Academy of Higher Education, Manipal, Karnataka, India. *; 2 *Department of Biostatistics, Vallabhbhai Patel Chest Institute, University of Delhi, Delhi, India. *

**Keywords:** Cervical cancer, miRNA, microRNA, prognosis, overall survival

## Abstract

**Aim:**

There is growing evidence for the possible use of microRNAs (miRNAs) in cancers as diagnostic as well as prognostic biomarkers in the present era of Personalized Medicine. The objective of the present systematic review and meta-analysis was to assess the prognostic role of microRNAs in uterine cervical cancers.

**Methods:**

A systematic review and meta-analysis was carried out searching electronic databases for published articles between January 2009 and August 2020 based on standard systematic review guidelines. Meta-analysis was performed by pooling the hazard ratio (HR) with 95% confidence interval (CI) to assess the prognostic value of deregulated miRNAs by the random-effects model.

**Results:**

In the present meta-analysis, the aberrant expression of 14 microRNAs in 1,526 uterine cervical cancer cases before definitive therapy from 14 case-control studies were assessed. The pooled HR of two miRNAs, miRNA-155 and miRNA-224 which were upregulated in cervical cancer tissues was 1.76 (95% CI 1.27-2.45) revealing significant association with overall poor survival. Meanwhile, the pooled HR was 1.53 (95% CI 0.94-2.94) when all the deregulated miRNAs in cervical cancer tissues were evaluated. The pooled HR of downregulated miRNAs was 1.46 (95% CI 0.81, 2.64). Meanwhile, the pooled HR of three upregulated miRNAs-425-5p, 196a, 205 in the serum sample was 1.37 (95% CI 0.45 -4.20).

**Conclusion:**

The downregulation of aberrant miRNAs was not associated with poor overall survival rates.

## Introduction

Cervical cancer is the fourth most common female cancer after breast cancer, colorectal and lung cancer (“Global Cancer Observatory,” n.d.). India is home to 453.02 million women aged 15 years and above who are at risk of uterine cervical carcinoma and is the second most common cancer among Indian women.(“India: Human Papillomavirus and Related Cancers, Fact Sheet 2018,” 2019) Despite being a preventable cancer by Human Papillomavirus (HPV) vaccination and cancer screening, one-fourth of the global burden of cervical cancers was contributed by India alone. Cervical cancer survival rates are highly variable across India and five-year age-standardized relative survival rate is much lower compared to other Asian countries such as China, South Korea, Singapore and Thailand (Bobdey et al., 2016).

The most common type of cervical cancer is squamous cell cancer constituting about 90% of uterine cervix cancers followed by adenocarcinoma comprising almost 10% (“Cervical Cancer Treatment (PDQ^®^)–Health Professional Version - National Cancer Institute,” 2020). In the absence of organized screening programs in India, most women present at advanced stages. Timely detection and management become almost impossible due to non-specific symptoms in the early stages and the absence of organized screening programmes in developing countries. The search for an efficient biomarker for early diagnosis is challenging owing to uncertain cellular and molecular mechanisms of cervical cancer oncogenesis. 

In the early 1990s, MicroRNAs (miRNAs) were discovered. MicroRNAs may function as oncogenes or tumor suppressors at various targets in human cancers leading to aberrant expressions. Recent studies carried out in the last decade reported dysregulation of some microRNAs in cervical cancer oncogenesis. MicroRNAs regulate about 30% of human genes (Garzon et al., 2006) and 50% of these genes are tumor-related or situated in fragile loci (Calin et al., 2004). Upregulation of miR-155 and miRNA-224 was observed to be poor prognostic factors in cervical cancers (Fang et al., 2016; Shen et al., 2013). Meanwhile downregulation of certain microRNAs was reported to imply poor prognosis in cervical cancers. These markers are stable in serum and are expressed in tissues. 

*Why it is important to do this review*

Several years of cytology screening in low- and middle-income countries have not led to a significant reduction in cervical cancer cases which is attributed to financial and technical hurdles in carrying out cytology as well as Human Papillomavirus (HPV) DNA testing, non-compliance for follow-ups and timely management of screen-positive women. Identifying efficient biomarkers for early detection and appropriate management of uterine cervical cancers which can be translated from bench to bedside is the need of the hour. We performed a systematic review and meta-analysis regarding the prognostic value of microRNAs in uterine cervical cancers. 

## Materials and Methods

A systematic review and meta-analysis of literature was performed searching electronic databases for published articles in English between January 2009—August 2020 based on standard systematic review guidelines(“Cochrane collaboration,” n.d.; “Systematic reviews, evidence synthesis - The Campbell Collaboration,” n.d.). The present rearch work comply with the Preferred Reporting Items of Systematic reviews Meta-Analyses (PRISMA) statement (Moher et al., 2009). The meta-analysis component was modified appropriately to evaluate the prognostic value of miRNAs in cervical cancer.

*Description of the condition*

Micro RNA or miRNA: MicroRNAs are short non-coding, single-stranded RNAs having 18-25 nucleotides length, which regulate gene expression at the post-transcriptional level through target messenger RNAs (mRNAs) and suppress translation or induce mRNAs degradation(Inui et al., 2010). Utilizing several molecular mechanisms, microRNAs play a vital role in the origin and progression of cancers, either as oncogenes or as tumor suppressors.

Prognosis: Prognosis is the chance of developing cervical cancer or possibilities of getting better after developing cancer. In the present research, the prognosis is reported in terms of overall survival rate due to cervical cancer.

Biomarker: Biomarker is a characteristic that can be measured implying normal biological processes, pathogenic processes or responses to an exposure or intervention (Group, 2017). A pathological or physiological process can be recognized by testing for a natural gene, enzyme or protein. 

*Study protocol*

An electronic search of PubMed/MEDLINE and SCOPUS was carried out using search terms such as miRNA OR microRNA AND cervical cancer AND “survival”. Retrospective case-control studies, employing quantitative real-time polymerase chain reaction (qPCR) for miRNA profiling in cervical squamous cell cancers were chosen for meta-analysis. The published articles in English since 2009 were explored and a manual library search for articles in peer-reviewed journals was performed. The references of retrieved articles were also examined to increase the search sensitivity. 

Inclusion process and criteria: Articles regarding *miRNA* expression in cancer tissue and matched adjacent normal cervical tissue samples or in serum samples of uterine cervix cancer cases belonging to clinical stages I-IV as per International Federation of Gynecology and Obstetrics (FOGO, 2018) classification (Bhatla et al., 2019) were included for the meta-analysis. Case-control studies employing quantitative real-time polymerase chain reaction (qPCR) for miRNA profiling were included. 

All the included studies categorized the samples into low expression and high expression groups based on the mean or median cutoff value of miRNA amplified. The overall survival rate was reported as hazard ratio (HR) with 95% confidence interval (CI) to evaluate the prognostic value of microRNAs. 

Exclusion criteria: Duplicate publications of the same survey and articles published in languages other than English were excluded. Studies reporting microRNA profiling by microarray were also excluded as these assays are less specific without absolute quantification. MicroRNA profiling extracted by bioinformatics assays, studies reporting only relative risk (RR) and articles with no information on histology of cancers were also excluded. 

*Data extraction*

 A validated proforma focusing on the first author, year of publication, region, sample size, histology, lymph node involvement, duration of follow up, miRNAs amplified, cut-off value, HPV status, and hazard ratio (HR) with 95% CI was used to extract the data.

**Figure 1.**
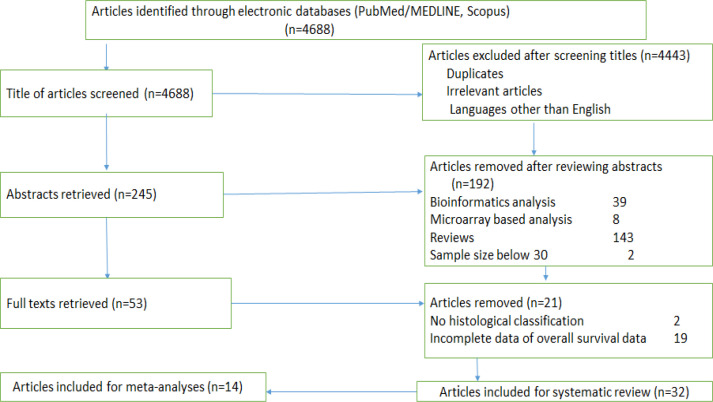
PRISMA Chart Ddetailing the Study Selection Process. The flow diagram demonstrating the number of studies identified, records screened, full-text articles evaluated for the eligibility, and the studies included in the systematic review and meta-analysis

In this systematic review, the three-stage selection process was carried out for the final inclusion of the studies. One reviewer assessed titles from 4688 records for the relevance for inclusion in the study. Studies applicable for the review were moved to the second stage and articles in languages other than English, irrelevant articles and duplicates were excluded (n= 4443). In the second stage, the abstracts of the studies (n=245) were obtained and two reviewers independently analyzed all the abstracts. All the non-relevant studies were rejected (n=192) and the remaining (n=53) studies were moved to the third stage. Full texts of studies (n=53) were retrieved which were again examined by two reviewers independently. Extracted data from full-text articles by the first reviewer was further reviewed by a second reviewer. Any difference of opinion was settled by discussion. A total of 32 studies were systematically reviewed. Detection of miRNA in cervical cancer cases from 14 case-control studies were included in the quantitative synthesis (meta-analysis). The study selection process is depicted in the PRISMA chart (Figure 1). The last date of the search was 23^rd ^August 2020. 

*Quality assessment*

Utilizing New Castle Ottawa scale (NOS), two reviewers independently and systematically assessed the quality of case-control studies (“Ottawa Hospital Research Institute,” n.d.) 

*Data analysis*

Considering the inclusion of possible confounding factors, articles reporting hazard ratio from multivariable Cox Regression analysis were included for meta-analysis. Adjusted hazard ratio (HR) along with 95% Confidence Interval (CI) from multivariable Cox Regression analysis were extracted from each of the 14 studies qualified for meta-analysis. These hazard ratios were synthesized in a meta-analysis to obtain a pooled HR with 95% CI to evaluate the prognostic effects of miRNAs on the survival of uterine cervical cancer. Meta-analysis was performed using a random-effects model in STATA 13.1 statistical software (College Station, Texas 77,845 USA).using the “Metan” package. Chi-square statistic and I-squared statistic were used as measures of statistical heterogeneity. The results of the meta-analysis are depicted in a forest plot, where the diamond represents the pooled estimate with 95% CI.

Forest plots were separately generated for miRNA expressed in tissue and serum samples. In both the meta-analyses a subgroup analysis was carried out based on the high or low expression of *microRNA*. The I^2^ statistic was computed to test the significance of potential heterogeneity. 

*Publication bias*

The publication bias was evaluated by Egger’s test which includes weighted linear regression with standardized effect estimate, the dependent variable and precision as the independent variable(“(PDF) MIDAS: Stata module for meta-analytical integration of diagnostic test accuracy studies | Ben Dwamena - Academia.edu,” n.d.). In the present study, loge HR was considered as the effect estimate and the precision was given as 1/standard error of loge HR. Weights were allotted by the inverse variance approach (1/variance of the effect estimate). A statistically significant bias coefficient gives evidence for the presence of publication bias.

## Results

*Included studies*

Through electronic search, full texts of 32 articles were reviewed systematically and 14 studies were included for quantitative synthesis. Characteristics of the included studies are shown in Table 1. All the fourteen studies were of good quality. A major proportion of studies were carried out in China, followed by Iran, Canada, and Korea regarding the prognostic role of microRNA in cervical cancer. Because of unsuccessful attempts at microRNA profiling, the Canadian study was excluded for quantitative synthesis (How et al., 2015). Another omitted study from Korea included only uterine cervical adenocarcinomas for analyzing the dysregulation of miRNA 363-p (Park et al., 2014). Meanwhile, as there was no histological classification, Iranian study also could not be included (Azizmohammadi et al., 2017). Two studies were not qualified for quantitative synthesis as less number of cases were included. Deregulation of microRNAs as per single study was also not considered for quantitative synthesis. 

The studies qualified for meta-analysis were all from China, the world’s most populous country. Studies reporting the prognostic value of 14 aberrant microRNAs in tissue or serum samples of 1,526 uterine cervical cancer cases prior chemoradiation were included for meta-analysis. Out of the fourteen qualified studies, ten articles reported *microRNA* expression in cervical cancer tissues. As shown in Table 1, four studies performed molecular quantification of microRNAs in serum samples of cervical squamous cell cancers (Jiang et al., 2017; Liu et al., 2015; Ma et al., 2014; Sun et al., 2017). We observed that most of the qualified studies for the meta-analysis used adjacent normal cervical tissues as controls. Four studies specified normal tissue as tissue retrieved 3 cm beyond the boundary of cervical cancer tissue (Shen et al.,2015; Zhang 2016;Wang 2015; Yang 2014). A recently published study by Liu et al enrolled patients who had undergone hysterectomy for benign conditions as controls (Liu et al., 2018) and all the remaining studies just mentioned that adjacent normal tissue was employed as controls.

The present systematic review observed that four studies reported the recurrence-free survival rate (RFS) apart from overall survival. Out of the 14 qualified studies, only two recent studies generated recurrence-free survival rate (RFS) from Kaplan Meier curves other than overall survival rate (OS) (Liu et al., 2018; Zhou et al., 2018) Deceased patients and cases having recurrence were found to have a lower *361-3p *expression when compared to healthy controls (Liu et al., 2018). Zhou et al., (2018) also reported lower RFS and overall survival with downregulation of miR1254.

*Quantitative synthesis*

**Figure 2:**
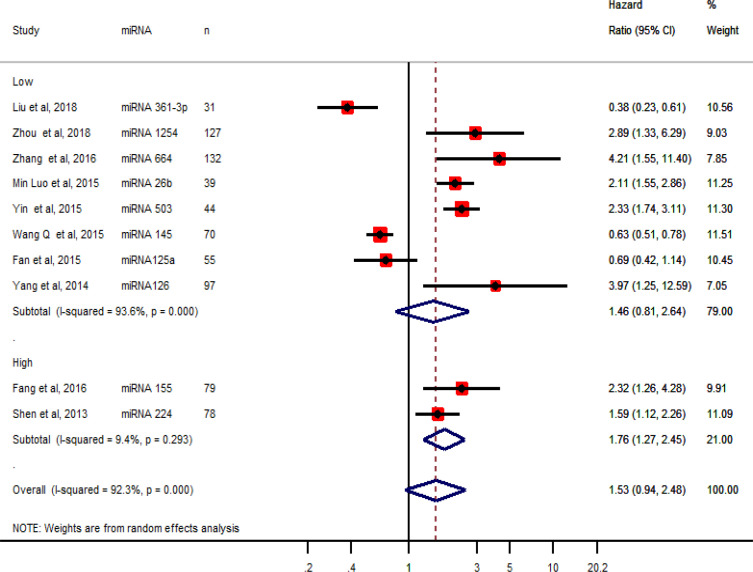
Forest Plot of the Pooled Hazard Ratio (HR) of microRNAs Expressed in Tissues with Overall Survival among Cervical Cancer Patients. The summary estimates were obtained by the random-effects model. The diamond data specifies pooled HRs. CI –confidence interval

As shown in Table 2, ten studies reported HPV DNA status along with *miRNA* expression. The HR with 95% CI (confidence interval) were pooled for miRNAs with low or high expression in tissue samples and analyzed by forest plot (Figure 2). The overall HR was 1.53 (95% CI 0.94-2.94) which was not associated with poor overall survival. Further analysis of HR with 95% CI for miRNAs with low expression demonstrated HR of 1.46 (95% CI 0.81, 2.64) with no significant association with overall poor survival. Meanwhile, the pooling of HR of two miRNAs, miRNA-155 and miRNA-224 which were upregulated in cervical cancer tissues resulted in pooled HR of 1.76 (95% CI 1.27-2.45) revealing significant association with overall poor survival (Figure 2). 

**Figure 3:**
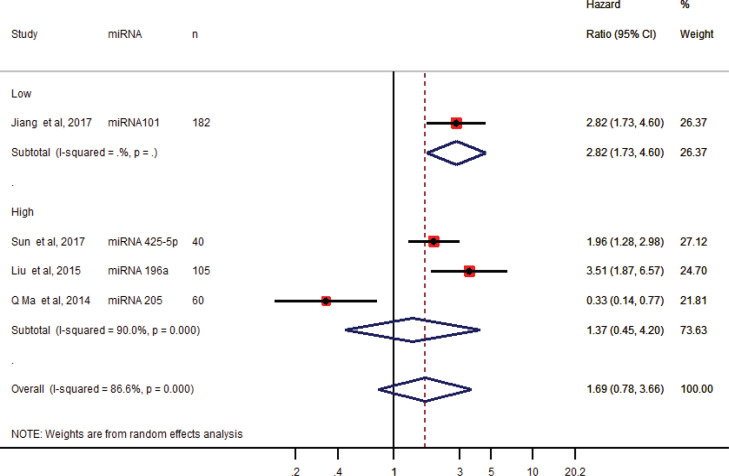
Forest Plot of the Pooled Hazard Ratio (HR) of microRNAs Expressed in Serum Samples of Cervical Cancer Cases with Overall Survival. The summary estimates were obtained by the random-effects model. The diamond data specifies pooled HRs. CI –confidence interval

The only one downregulated microRNA in the serum sample, miRNA-101 was associated with poor overall survival (Jiang et al., 2017). The pooled HR of three upregulated miRNAs-196a, 215, 425-5p in serum samples was 1.37 (95% CI 0.45 -4.20) implying a lack of significant association with overall poor survival (Figure 3). Pooling the HR from all the four studies in serum samples had no significant association with poor overall survival as the pooled HR was 1.69 (95% CI 0.78-3.66). 

As the I^2^ ≥ 50%, indicating considerable heterogeneity, the random-effects model was adopted for pooling. Compared to previous meta-analyses considerable heterogeneity was observed as more number of studies with different miRNA profiles were pooled. 

*Publication bias*

The p-value for the bias coefficient was not statistically significant (Tables 3 and 4). Hence there was no evidence of publication bias.

**Table 1:** Main Characteristics of Qualified Studies for Meta-Analysis (n=14)

	Reference (years)	Country	Age (years)	Number (n)	Sample	Histology		Lymph node metastasis		Quality of studies NOS
						squamous	Non-squamous	No	Yes	Good
1	Liu 2018 ^24^	China	Mean 46.3	31	Snap frozen tissue	31	0	Not available	Not available	Good
2	Zhou 2018 ^255^	China	< 65- 76 ≥ 65-105	181	Snap frozen Tissue	74	107	114	67	Good
3	Zhang 2016 ^27^	China	< 65- 81 ≥ 65-105	186	Snap frozen tissue	137	49	82	104	Good
4	Fang et al 2016 ^7^	China	<50-63 ≥50-66	129	Snap frozen tissue	86	43	78	51	Good
5	Min Luo et al 2015^2^	China	<50- 35 ≥50-53	88	Snap frozen tissue	62	26	42	46	Good
6	Yin et al 2015 ^29^	China	<55-37 ≥55- 59	96	Flash frozen tissue	67	29	43	53	Good
7	Wang Q 2015 ^26^	China	Median ^50^	114	Snap frozen tissue	114	0	43	71	Good
8	Fan et al 2015 ^30^	China	Mean 54.4±9.9	55	Snap frozen tissue	50	5	26	29	Good
9	Yang et al 2014 ^31^	China	< 65- 56 ≥ 65- 77	133	Snap frozen tissue	101	32	70	63	Good
10	Shen et al 2013^ 8^	China	Median 50	126	Frozen tissue	126	0	44	82	Good
11	Jiang 2017 ^23^	China	Mean 53±9	182	serum	139	43	145	37	Good
12	Sun 2017 ^22^	China	≤60-22 >60-18	40	serum	10	30	11	29	Good
13	Liu 2015 ^21^	China	<50-64 ≥50 41	105	serum	84	21	68	37	Good
14	Q Ma 2014 ^20^	China	Median 51	60	serum	60	0	22	38	Good

NOS, New castle Ottawa scale

**Table 2:** Prognostic Value of Micro RNAs in Uterine Cervical Cancer (n=14)

	Author (year)	Follow up (months)	miRNA	HPV status		HR (95% CI) Multivariate analysis	Potential targets	Expression Associates with poor prognosis
				(+)	(-)			
1	Liu 2018 ^24^	24	miRNA- 361-3p	---	----	0.377 (0.233-0.608)	SOST, MTA1, TFRC,YAP1	Low
2	Zhou 2018 ^25^	60	miRNA-1254	127	54	2.889 (1.452-6.886)	N4BP3	Low
3	Zhang 2016 ^27^	70	miRNA-664	132	54	4.21 (2.36-17.32)	c-Kit	Low
4	Fang et al 2016 ^7^	60	miRNA-155	79	50	2.32 (1.259-4.276)	LKB1 SMAD2, CCND,	High
5	Min Luo et al 2015^28^	74 (mean)	miRNA-26b	39	49	2.107(1.744-3.211)	JAG1	Low
6	Yin et al 2015 ^29^	60	miRNA-503	44	52	2.327(1.922-3.436)	CCND1	Low
7	Wang Q 2015 ^26^	47 (median)	miRNA-145	70	44	0.63 (0.54–0.83)	IRS-1, HLTF	Low
8	Fan et al 2015 ^30^	42	MiRNA-125a	---	-----	0.691(0.418-1.141)	STAT 3	Low
9	Yang et al 2014 ^31^	60	miRNA-126	97	36	3.97(2.01-20.22)	ZEB 1	Low
10	Shen et al 2013 ^8^	Median 51.9	miRNA-224	78	48	1.59(1.12-2.26)	PTX3	High
11	Jiang 2017 ^23^	60	miRNA-101	-	-	2.820 (1.473–3.925)	COX-2	Low
12	Sun 2017 ^22^	60	miRNA-425-5p	-	-	1.957 (1.224-2.843)	AIFM1	High
13	Liu 2015 ^21^	80	miRNA-196a	92	13	3.51 (1.961–6.874)	HOXC8	High
14	Q Ma 2014^ 20^	60	miRNA-205	54	6	0.33 (0.14-0.76)	CYR61 and CTGF	High

**Table 3:** Result of Egger's Test of Forest Plot Regarding the Pooled Hazard Ratio (HR) of microRNAs Expressed in Tissues of Cervical Cancer Cases

Coefficient	Estimate (95%CI)	P-value
Slope	-0.2905 (-1.4353, 0.8544)	0.575
Bias	2.7114 (-3.0859, 8.5087)	0.312

**Table 4:** Results of Egger’s Test of Forest Plot Regarding the Pooled Hazard Ratio (HR) of microRNAs Expressed in Serum Samples of Cervical Cancer Cases

Coefficient	Estimate (95%CI)	P-value
Slope	2.2598 (-4.2961, 8.8157)	0.276
Bias	-5.8059 (-29.6182, 18.0063)	0.404

## Discussion

The preservation of certain miRNAs-361-3p, 1254, 664, 155, 26b, 503,145,125a, 126, 224, 101, 425-5p, 196a, 205 in cervical cancer was observed to be associated with improved overall survival and better prognosis (Fan et al., 2015; Fang et al., 2016; Jiang et al., 2017; Liu et al., 2015, 2018; Luo et al., 2015; Ma et al., 2014; Shen et al., 2013; Sun et al., 2017; Wang et al., 2015; Yang et al., 2014; Yin et al., 2015; Zhang et al., 2016; Zhou et al., 2018). The present meta-analysis observed upregulation of two micro RNAs-155 and -224 in tissues was related to poor prognosis and reduced overall survival. Upregulation of oncogenic miRNA-155 and miRNA-224 was noted in HPV positive cervical cancers (Fang et al.,2016; Shen et al.,2013). Increased expression of *miRNA-224* was reported to be an independent predictor of poor prognosis, lymph node metastasis and shorter overall survival (Shen et al., 2013). MicroRNA-155 is an oncomir which promotes malignant transformation in many human cancers such as cervical cancer, lung cancer, leukemia and breast cancer (Jiang et al., 2010; Lao et al., 2014; Marcucci et al., 2013; Yang et al., 2013). Tumor suppressor gene, LKB1 encoding serine/threonine kinase was identified as the potential target of miRNA-155 based on bioinformatics analysis(Zhang et al., 2014). In cervical cancer cells LKB1 gene was reported to be downregulated (Zhang et al., 2014). PTX3 was reported to be the target gene for miRNA-224 when analyzed by bioinformatics as well as dual-luciferase reporter assay. Overexpresssion of miRNA-224 and silencing or under expression of *PTX3* was related to aggressive progression of cervical cancers (Yu et al., 2018).These observations may facilitate the development of potential therapeutic agents against cervical cancer in future. Deregulated expression of *microRNAs* leads to silencing of the target gene which may be affecting the response to various chemothrepeutic agents (Wu and Xiao, 2009). 

In the present meta-analysis, downregulated microRNAs were not found to be influencing the overall survival. This was not in agreement with the meta-analysis by Dai et al., (2016) which pooled HR from nineteen studies. Dai et al., (201) observed the association of low miRNA with poor survival. Another meta-analysis of seven studies by Chen et al., (2018) observed that poor survival was associated with low levels of miRNA-125, miRNA-145, and a high level of miRNA-196 in tissue samples. We observed vast variation in the histological types of cancer in the studies included by Chen et al., (2018) as well as Dai et al., (2018) which pooled HR from studies carried out in serum and tissue samples in a single meta-analysis. Compared to serum, aberrant *miRNA* expression in tissue is more specific for assessing the prognostic value in site-specific cancers. Studies with histological classification were included in the present meta-analysis and except one study squamous cell carcinoma cases was the predominant type in all the 13 articles (Table 1). Separate pooled hazard ratios with 95% CI were synthesized for miRNAs amplified in tissue and serum samples. 

The causal role of Human Papillomavirus (HPV) in cervical cancer is well proven and aberrant *miRNA* expression in cervical cancers was attributed to persistent high-risk HPV infection (Zheng and Wang, 2011). Limited number of reviewed studies reported Human Papillomavirus association along with miRNA profiling as in Table 2. A recent study by Liu observed decreased *miRNA-361-3p* expression in high-risk HPV DNA positive cervical tissues when compared to normal tissues without HPV infection. However, a significant difference in *miRNA-361-3p *expression was not observed between cervical cancer tissue and normal tissue (Liu et al., 2018). Downregulation of tumor suppressor *miRNA-145* was observed in HPV positive uterine cervical squamous cell cancers with vascular invasion (Wang et al., 2015). There was no significant correlation between HPV positivity and certain tumor suppressors such as *miRNA-1254*, and *miRNA-664 *(Zhou et al.,2018; Zhang et al.,2016). Chromosomal instability caused by HPV is attributed to upregulation of microRNA-9 in uterine cervical cancers (Korzeniewski et al., 2011; Liu et al., 2014). Fan et al., (2015) reported HPV-induced p53 inactivation resulting in microRNA-125a downregulation. 

*Strengths of the study*

Separate meta-analyses were performed for tissue and serum samples. Subgroup analysis was also performed for differential *miRNA* expression. Estimates of HR were obtained only from multivariable Cox regression analysis. There was uniformity in the tissue samples preserved for the study in the present meta-analysis.

There were several limitations of our meta-analysis. Except for one study, all the included studies incorporated adjacent normal cervical tissue from cervical cancer cases as controls. There was ambiguity regarding the microscopic examination for malignant changes. There was no uniform cut-off levels even though median or mean cut off values were used in the included studies. Due to the scarcity of published research on the microRNAs, we had to pool different microRNAs for evaluating the prognostic role. However, miRNAs reported to be deranged in at least two research publications were selected for the meta-analysis. Amongst the qualified studies miRNA-155 and -224 were upregulated in tissue samples which was significantly associated with poor overall survival in cervical cancers. This should be interpreted with caution as the pooled HR was generated from only two published articles. 

*Recommendations*

Future research including cervical tissues from women with no cervical cancers as controls is needed. Research work on upregulated microRNAs has to be undertaken among cervical cancer cases wordwide. Large prospective studies are needed to assess the prognostic value of microRNAs in cervical cancer. 

In conclusion, the downregulation of miRNAs was not associated with poor overall survival rates in cervical cancer. 

## Author Contribution Statement

SS and NR and conceptualised the study and developed the research protocol; SS and NR identified articles for full-text review, extracted data from studies, and matched inclusion criteria. N R did the statistical analyses. SS drafted the study. Both the authors approved the final study, and NR agreed to be accountable for all aspects of the work in ensuring that questions related to the integrity of any part of the work are appropriately investigated and resolved.
